# Interaction of renin-angiotensin system gene polymorphisms with hypertension in Chinese patients with type 1 diabetes and retinopathy

**DOI:** 10.18632/oncotarget.24100

**Published:** 2018-01-09

**Authors:** Yong-Chao Qiao, Yan-Hong Pan, Yan Xu, Xiao-Xi Zhang, Hai-Lu Zhao

**Affiliations:** ^1^ Department of Immunology, Xiangya School of Medicine, Central South University, Changsha, Hunan, China; ^2^ Center for Diabetic Systems Medicine, Guangxi Key Laboratory of Excellence, Guilin Medical University, Guilin, Guangxi, China; ^3^ Department of Laboratory Medicine, Hunan Aerospace Hospital, Changsha, Hunan, China; ^4^ Department of Immunology, Faculty of Basic Medicine, Guilin Medical University, Guilin, Guangxi, China

**Keywords:** type 1 diabetes mellitus, gene polymorphisms, genotype, diabetic retinopathy, renin-angiotensin system

## Abstract

**Background:**

The objective of this research was to investigate the interaction of RAS gene polymorphisms in Chinese patients with type 1 diabetes mellitus (T1DM) and diabetic retinopathy (DR).

**Methods:**

Genomic DNA was extracted from peripheral blood leukocytes and genotyping for the angiotensin converting enzyme (ACE) gene I/D and angiotensinogen (AGT) gene M/T polymorphisms was performed using the polymerase chain reaction method. 311 T1DM patients were recruited for the assessment of ACE and AGT polymorphisms relating to DR.

**Results:**

Compared with the diabetic non-retinopathy (DNR) patients, DR patients had lower proportion of diabetic nephropathy (*p*<0.001) and M allele (*p*=0.013). Intriguingly, the frequency D allele (*p*=0.035) was lower in DR patients with hypertension, as well as DD (*p*=0.003) and DI genotype (*p*=0.012) in DR patients with normal blood pressure after multiple tests with Bonferroni correction, but D allele (*p*=0.025) displayed higher in normotensive patients with T1DM. Logistic regression analyses indicated that no significant relationship existed about the genotype and allele polymorphisms with the progress of DR after adjusting for confounding factors.

**Conclusions:**

Interaction of hypertension and the RAS gene polymorphisms might have a role in the DR development in Chinese T1DM patients.

## INTRODUCTION

Diabetic retinopathy (DR), one of the most damaging microvascular complications of diabetes mellitus which occurs in both type 1 and type 2 diabetes mellitus (T1DM and T2DM) [1]. Previous epidemiological studies have indicated that all T1DM patients and nearly about 60% of T2DM patients will develop this complication [1]. Risk factors leading to DR are uncertain. Several lines of evidence suggest that a relationship between DR and gene polymorphisms of various components of the renin-angiotensin system (RAS) may potentially alter susceptibility to DR in diabetes [2, 3]. RAS consists of renin, angiotensinogen (AGT), angiotensin converting enzyme (ACE), ACE2, angiotensin II type 1 receptor (AT1R) and AT2R [4, 5]. ACE is a component of the RAS which plays an important role in the regulation of systemic and renal circulation by converting angiotensin I into vasoconstrictor molecule angiotensin II [6]. AGT is converted to angiotensin I by renin, and subsequently into angiotensin II by ACE [7]. For decades, a number of studies have reported about the relationship between ACE, AGT gene polymorphisms and DR [1, 4, 6, 8–12], however, the conclusions remain inconsistent. Most of the work focused on the role of RAS in T2DM patients, but few efforts devote to the pathogenesis of T1DM about insulin resistance in clinical studies. Our previous works have proved that an association exists between diabetic glomerulosclerosis with immunoreactivity and RAS gene polymorphisms [13]. Hereby, we explore the interaction of ACE and AGT gene polymorphisms with known risk factors such as hypertension for the development of DR in Chinese T1DM patients.

## RESULTS

### Characteristics of the patient samples and clinical findings

In this cross-sectional study, a total of 311 patients with T1DM were enrolled: 92 subjects with DR and 219 diabetic subjects without retinopathy. In fact, 155 patients (54 with DR, 101 with diabetic non-retinopathy, DNR) were determined with ACE genotypes and 133 (48 with DR, 85 with DNR) with AGT genotypes due to the loss of blood samples. Clinical characteristics of all subjects are displayed in Table 1. Compared with DNR patients, the DR patients were older (*p*<0.001) and had lower frequency of male sex (*p*=0.020), older at onset (*p*=0.017), longer duration of known diabetes (*p*<0.001), higher proportion of hypertension (*p*=0.020), higher level of albuminuria (*p*<0.001), plasma urea (*p*<0.001), plasma creatinine (*p*=0.029), ACR (*p*<0.001), AER (*p*<0.001), and systolic blood pressure (SBP, *p*<0.001).

**Table 1:** Clinical characteristic of 311 patients with type 1 diabetes

	Total	DNR	DR	*p*
n	311	219	92	-
Age (years)	46.27±16.53	43.93±16.30	51.90±15.76	<0.001^†^
Male (%)	48.71	52.97	38.46	0.020^‡^
Smoker (%)	14.29	13.76	15.87	0.678^‡^
Age at onset (years)	39.58±17.45	38.06±17.18	44.08±17.60	0.017^‡^
Duration (years)	5.00(1.00-10.00)	4.00(1.00-9.00)	10.00(4.25-13.00)	<0.001^§^
BMI (kg/m^2^)	24.05±4.07	24.09±4.30	23.97±3.46	0.825^†^
Waist (cm)	81.15±11.26	80.66±11.79	82.45±9.71	0.194^†^
Hip (cm)	94.90±7.67	95.25±8.00	93.97±6.67	0.213^†^
C-peptide (mg/L)	1.70(0.77-2.49)	1.96(1.09-3.66)	1.51(0.74-2.29)	0.533^§^
Hypertension (%)	36.54	32.39	46.6	0.020^‡^
SBP (mmHg)	131.64±24.36	128.30±23.44	139.72±24.78	<0.001^†^
DBP (mmHg)	77.01±12.81	76.43±12.41	78.41±13.69	0.223^†^
HbA_1c_ (%)	7.71±2.08	7.72±2.16	7.70±1.87	0.941^†^
FPG (mmol/L)	7.71±2.08	7.72±2.16	7.70±1.88	0.949^†^
TG (mmol/L)	1.15(0.69-1.79)	1.11(0.63-1.70)	1.13(0.73-1.79)	0.469^§^
TC (mmol/L)	5.28±1.28	5.20±1.15	5.49±1.55	0.074^†^
LDL-C (mmol/L)	3.10(2.58-3.80)	3.10(2.50-3.85)	3.15(2.60-3.85)	0.302^§^
HDL-C (mmol/L)	1.27(1.27-1.56)	1.32(1.04-1.73)	1.23(1.02-1.45)	0.108^§^
Albuminuria	2.00(2.00-6.00)	2.00(2.00-4.00)	6.00(6.00-14.00)	<0.001^§^
Plasma urea (mmol/L)	5.40(4.50-6.60)	5.30(4.35-6.30)	6.10(5.13-7.30)	<0.001^§^
Plasma creatinine (μmol/L)	75.00(61.00-93.00)	73.00(73.00-89.00)	81.00(61.25-104.00)	0.029^§^
ACR (mg/g)	1.64(0.82-6.83)	1.25(0.72-3.10)	6.70(2.07-59.81)	<0.001^§^
AER (μg/min)	9.50(4.83-38.85)	8.18(8.18-22.07)	29.12(7.17-385.23)	<0.001^§^
Diabetes	217	175(92.59)	42(65.63)	<0.001^†^
Diabetic nephropathy	36	14(7.41)	22(34.37)	
Total	253	189(100)	64(100)	

DNR, diabetic non-retinopathy; DR, diabetic retinopathy; Data are shown as means ± SD, median (inter-quartile range) or percent. ^†^Derived from the *t* test. ^‡^Derived from the *χ*^*2*^ test. ^§^Derived from the Mann-Whitney *U* test.

### The relation between ACE, AGT gene polymorphisms and DR

Table 2 shows the genotype and allele frequencies of the ACE and AGT. Genotype frequencies in all groups are all in accordance with the Hardy-Weinberg equilibrium (all *p*>0.05). No significant association was found between the groups about the frequency of ACE (DD vs. DI vs. II, *χ*^*2*^=2.320, *p*=0.313) and AGT gene polymorphisms (MM vs. MT vs. TT, *χ*^*2*^=4.154, *p*=0.125), as well as the frequency of allele (D vs. I, *χ*^*2*^=0.839, *p*=0.403) except M vs. T (*χ*^*2*^=6.159, *p*=0.013) (Table 2).

**Table 2:** Correlation of RAS polymorphisms with diabetic retinopathy after matching for age, duration of known diabetes, and blood pressure in type 1 diabetes

Genotype and allele		Total	DNR	DR	Comparison	p^*^	Power value
ACE	DD	44	25(24.75)	19(35.19)	DD VS. DI+II	0.170	0.284
	DI	56	40(39.60)	16(29.63)	DI VS. DD+II	0.218	0.228
	II	55	36(35.64)	19(35.19)	II VS. DD+DI	0.955	0.050
	Total	155	101(100)	54(100)	DD VS. DI VS. II	0.313	-
	D	144	90(44.55)	54(50.00)	D VS. I	0.403	0.150
	I	166	112(55.45)	54(50.00)			
	Total	310	202(100)	108(100)			
AGT	MM	25	12(14.12)	13(27.08)	MM VS. MT+TT	0.066	0.461
	MT	28	17(20.00)	11(22.92)	MT VS. MM+TT	0.692	0.073
	TT	80	56(65.88)	24(50.00)	TT VS. MM+MT	0.072	0.440
	Total	133	85(100)	48(100)	MM VS. MT VS. TT	0.125	-
	M	78	41(24.12)	37(38.54)	M VS. T	0.013	0.695
	T	188	129(75.88)	59(61.46)			
	Total	266	170(100)	96(100)			

Data are shown as n (percent). ^*^Derived from the χ2 test.

Considering the confounding risk factors including BMI, albuminuria, dyslipidemia and hypertension and so on, binary logistic regression analyses were used to estimate the odds ratios (ORs) for the incidence of retinopathy in diabetes patients. The result of binary logistic regression (groups [DNR vs. DR] as the dependent variable and age, age on set, duration of diabetes, BMI, hypertension [%], HbA_1c_, FPG, TG, TC, LDL-C, HDL-C, albuminuria, plasma urea, plasma creatinine, ACR, AER, and ACE, AGT genotype as covariates) indicated that no significant relationship was found about the ACE and AGT gene polymorphisms with diabetic retinopathy after adjusting for confounding factors (Table 3).

**Table 3:** Odds ratios of ACE/AGT genotypes for diabetic retinopathy in Chinese patients with type 1 diabetes

		Model 1^†^		Model 2^‡^		Model 3^§^		Model 4^¶^	
		ORs (95% CI)	*P*	ORs (95% CI)	*P*	ORs (95% CI)	*P*	ORs (95% CI)	*P*
ACE	DD	0.300(0.013-7.013)	0.454	0.189(0.007-4.884)	0.315	0.341(0.010-11.655)	0.550	0.480(0.009-25.018)	0.716
	DI	1.041(0.276-3.925)	0.953	1.235(0.309-4.939)	0.766	1.180(0.245-5.689)	0.837	1.345(0.247-7.330)	0.732
	II	1	-	1	-	1	-	1	-
AGT	MM	10.133(0.715-143.593)	0.087	13.220(0.816-214.219)	0.069	18.333(1.001-335.817)	0.050	22.289(0.804-617.777)	0.067
	MT	1.364(0.319-5.832)	0.675	1.149(0.251-5.257)	0.858	1.043(0.184-5.899)	0.962	0.980(0.150-6.385)	0.983
	TT	1	-	1	-	1	-	1	-

^†^Model 1 adjusted for Age, Sex, Age onset, Duration of diabetes. ^‡^Model 2 adjusted for model 1 + BMI, hypertension. ^§^Model 3 adjusted for model 2 + HbA1c, FPG, TG, TC, Albuminuria. ^¶^Model 4 adjusted for model 3 + Plasma urea, Plasma creatinine, ACR, AER.

### Concordance between DR and diabetic nephropathy

ACR and AER were used to define microalbuminuria and macroalbuminuria which was considered as diabetic nephropathy. Compared with DNR, the DR patients had significantly lower proportion of diabetic nephropathy (*χ*^*2*^=28.489, *p*<0.001), indicating significant concordance between DR and diabetic nephropathy (Table 1).

### The risk factor about hypertension

Hypertension maybe an important risk factor for the incidence of retinopathy in diabetes patients and in order to explore the influence, the patients was divided into hypertensive group and normotensive group. Intriguingly, as shown in Table 4, compared with DM controls, significant lower frequency about D allele and DD genotype was related to DR in the hypertensive group (D vs. I, *χ*^*2*^=4.427, *p*=0.035) and in normotensive group (DD vs. DI+II, *χ*^*2*^=11.232, *p*=0.003; DI vs. DD+II, χ^2^=8.177, *p*=0.012) after multiple tests with Bonferroni correction, and that higher D allele frequency was found in normotensive group (D vs. I, *χ*^*2*^=4.997, *p*=0.025). Otherwise, logistic regression analyses displayed that no significant relationship existed about the genotype and allele polymorphisms with the progress of diabetic retinopathy after adjusting for confounding factors (all *p*>0.05). Most values of power calculation (Tables 2 and 4) based on the sample size were lower than 0.8, indicating caution to interpret the results.

**Table 4:** Correlation of RAS polymorphisms with hypertension and diabetic retinopathy in patients with type 1 diabetes

Genotype and allele		Comparison	Hypertensive patients with type 1 diabetes					Normotensive patients with type 1 diabetes				
			Total	DNR	DR	p^*^	Power value	Total	DNR	DR	p^*^	Power value
ACE	DD	DD VS. DI+II	18	13(56.52)	5(23.81)	0.081^†^	0.608	18	7(9.72)	11(37.93)	0.003^†^	0.002
	DI	DI VS. DD+II	12	4(17.39)	8(38.10)	0.124	0.334	44	36(50.00)	8(27.59)	0.012^†^	0.552
	II	II VS. DD+DI	14	6(26.09)	8(38.10)	0.393	0.135	39	29(40.28)	10(34.48)	0.588	0.083
	Total	DD VS. DI VS. II	44	23(100)	21(100)	0.078	-	101	72(100)	29(100)	0.003	-
	D	D VS. I	48	30(65.22)	18(42.86)	0.035	0.550	80	50(34.72)	30(51.72)	0.025	0.608
	I		40	16(34.78)	24(57.14)			122	94(65.28)	28(48.28)		
	Total		88	46(100)	42(100)			202	144(100)	58(100)		
AGT	MM	MM VS. MT+TT	10	4(20.00)	6(30.00)	0.465	0.114	13	7(11.86)	6(24.00)	0.160	0.308
	MT	MT VS. MM+TT	6	2(10.00)	4(20.00)	0.661^‡^	0.138	20	14(23.73)	6(24.00)	0.979	0.049
	TT	TT VS. MM+MT	24	14(70.00)	10(50.00)	0.197	0.245	51	38(64.41)	13(52.00)	0.287	0.190
	Total	MM VS. MT VS. TT	40	20(100)	20(100)	0.420	-	84	59(100)	25(100)	0.347	-
	M	M VS. T	26	10(25.00)	16(40.00)	0.152	0.294	46	28(23.73)	18(36.00)	0.103	0.376
	T		54	30(75.00)	24(60.00)			122	90(76.27)	32(64.00)		
	Total		80	40(100)	40(100)			168	118(100)	50(100)		

Data are shown as n (percent). ^*^Derived from the *χ*^*2*^ test. ^†^Multiple tests with Bonferroni correction. ^‡^Derived from the Fisher’s exact test. Hypertension was defined as an average blood pressure ≥ 140/90 mm Hg or on blood pressure-lowering treatment.

## DISCUSSION

The frequency of individuals having diabetes is rapidly increasing, with an attendant toll of complications, including DR. Although the underlying mechanisms remain elusive, genetic susceptibility as a key to types of diabetes (I and II) is increasingly recognized for its contribution to diabetic complications. Several studies have provided evidence that multiple factors determine the risk of DR. Some factors may be metabolic, others may be genetic systemic conditions, or local factors in the eye itself may be responsible. Otherwise, such as hyperglycemia and hypertension [14], dyslipidemia [15] and diabetic nephropathy [16] have been shown to have significant relationship with the development and progression of DR. Beyond of above factors, genetic gene polymorphisms obtained more attentions about the pathophysiological process leading to proliferative retinopathy in recent studies, such as ACE I/D, AGT M/T gene.

In this study, we reported that the relationship between ACE, AGT gene polymorphism and T1DM subjects with retinopathy was not significant and the results were still consistent after adjusting for confounding factors. Our negative findings were consistent with most of previous studies [8, 17]. These findings indicate that the suggested role of genetics in predisposition to diabetic retinopathy is unlikely to be mediated through differences in the DNA sequence of the ACE, AGT gene, and that I/D, M/T polymorphism of this gene is not a useful marker to assess susceptibility to diabetic retinopathy. Otherwise, we found that the DD genotype and D allele were strongly associated with hypertension. The results were also consistent with a previous study. Frost, D [18] also suggested that young T1DM patients exhibited a relationship between the prevalence of hypertension and frequency of the D allele mediated by the level of ACE. The high level of renin and ACE in diabetic patients could lead to an excess of angiotensin II in the eyes, and elevate local intravascular blood pressure to hasten the development of retinopathy [19]. In addition, the higher level of ACE in patients with proliferative retinopathy indicates a potential role of ACE in retinal vascular damage for DR. ACE levels are under genetic control and the polymorphism of ACE gene may contribute to the variability of ACE plasma levels, as evident by the finding that homozygosity for the D allele is strongly linked to the highest plasma concentrations [20]. Saddick, S. Y. et al. evaluated the relationship between ACE gene polymorphism and the risk of patients with mild gestational hyperglycemia and also found that ACE genotypes were not associated in Saudi population [21].

In this study, we also found no significant relationship existed between AGT M235T gene polymorphisms and DR except the frequency of M/T allele. Rahimi, Z [4] and Gutierrez, C [22] found the gene polymorphism might not affect the risk of DM, which partly supported our findings. Otherwise, the presence of AGT 235T allele is associated with increased plasma level of AGT [4]. A relationship between the AGT gene, AGT levels, and insulin sensitivity in humans has been suggested with an association between AGT M235T polymorphism and increased insulin resistance [23]. Though the AGT M235T polymorphism has been complicated in the pathogenesis of arterial hypertension [24], it has not been associated with hypertension in T1DM [25] and T2DM patients. Base on the above, we supposed that the reason of our findings was that the rate-limiting step of the RAS was the enzymatic cleavage of AGT by renin and conversion of AGT to Ang II but not the level of AGT. Though the significant difference was found about M/T allele in our outcome, multiply complicated risk factors should be considered when illustrated the relationship between AGT polymorphism and Chinese T1DM patients with retinopathy.

It should be noticed that there were several limitations that need to be taken into consideration when interpreting our findings. Firstly, the levels of ACE and AGT were not detected because of the small volume of blood samples which maybe have more precise explains for the results. Secondly, due to the small sample size and lack sufficient data, we were unable to perform further analysis about the correlation between RAS gene polymorphism and DR according to glycemic index, triglyceride, HbA1c, duration of diabetes and so on. Thirdly, the ACE2, AT1R and AT2R gene polymorphisms of RAS system not further analyzed maybe play an important role in the relationship which maybe influences the findings. Lastly, the lower statistical power, some unmatched baseline data and the small sample size may be the possible explanation for the negative findings in this study. Undoubtedly, though the limitations were inevitable in our study, the strength of our research based on the cross-sectional study had a power to get a more precise estimation and evidence for the further study.

## MATERIALS AND METHODS

### Subjects and clinical measurements

In this cross-sectional clinic-genetic correlation study, a total of 311 T1DM Chinese patients were stratified into DR group (n=92) and DNR group (n=219). All the patients gave informed consent and the study was approved by the ethics committee of Guilin Medical University Affiliated Hospital (GLMC191211HL). There was no ethnicity bias in the data based on that all the patients who participated in this study belonged to the same ethnic group. Only newly diagnosed diabetes patients were included in this study and patients on treatment with blood pressure-lowering drugs including RAS blockers and anti-diabetic drug were excluded. The patients sampled from the database of the university affiliated hospital before 2014 and clinical diagnosis of DR was according to the Ophthalmology and Endocrinology Clinics. All patients were initially diagnosed for T1DM by a qualified endocrinologist. The diagnosis of diabetes was based upon the American Diabetes Association (ADA) criteria [26]. Patients were subjected to detailed eye examination with ophthalmoscopy and funduscopy and fundus photography to assess retinopathy. In addition to a thorough physical examination, we examined the following variables for each patient: body mass index (BMI), fasting plasma glucose (FPG), glycated haemoglobin (HbA_1c_), lipid profile (triglyceride [TG], total cholesterol [TC], high-density lipoprotein cholesterol [HDL-C], and low-density lipoprotein cholesterol [LDL-C]), albumin creatinine ratio (ACR) and albumin excretion rate (AER), as described in our previous report [13].

### ACE and AGT genotyping

Genomic DNA was extracted from peripheral blood leukocytes by protocols reported in our previous study [27]. The ACE gene I/D and AGT gene M/T polymorphisms were accessed through primers flanking the polymorphic region of intron 16 and 354 bp of exon 2, respectively. The standard primers of ACE genotype (I/D) and AGT genotype (M/T) in our previous work were used in this study [13]. The primers of ACE I/D polymorphism was 5’-CTGGAGACCACTCCCATCCTTTCT-3’ and 5’-GATGTGGCCATCACATTCGTCAGAT-3’, and the primers of AGT M/T gene polymorphism was 5’-CAGGGTGCTGTCCACACTGGACCCC-3’ and 5’-CCGTTTGTGCAGGGCCTGGCTCTCT-3’, respectively [13]. A 490 bp fragment (ACE I allele) and/or a 190 bp fragment (ACE D allele) and a 266 bp fragment (AGT M235 allele) and/or a 303 bp fragment (AGT 235T allele) were revealed by polymerase chain reaction (PCR) amplification, as described in detail previously [13]. Genotyping for the ACE gene I/D and AGT gene M/T polymorphisms was performed using the PCR method as our previous report [27].

### Definitions and calculations

An average blood pressure ≥ 140/90 mm Hg at least three different occasions at rest state or by the presence of antihypertensive treatment was defined as hypertension [13]. Normoalbuminuria, microalbuminuria and macroalbuminuria was defined according to urine AER or ACR [13] and the patients with microalbuminuria and macroalbuminuria were considered as diabetic nephropathy.

### Statistical analysis

Mean ± standard deviation (SD), median (inter-quartile range) or percentages were expressed for the data in this study as appropriate. If the genotypes and alleles were in Hardy-Weinberg equilibrium, the *χ*^*2*^ test was performed to compare the genotype distribution of each polymorphism and also was used to find out differences between groups for categorical variable, and if the total sample size is less than 40 or single sample size is less than 4, we use the Fisher’s exact test to explore the difference. Student’s *t*-test and one-way ANOVA in normal distribution were used to analysis the differences in continuous variables, otherwise, Mann-Whitney *U* test was performed for abnormal distribution. Logistical regression models were used to analyze the influence of known or suspected risk factors on developing DR after adjusting for various factors. The statistical power calculation was conducted through the PASS 11.0 software and the power value more than 0.8 displayed a high statistical power in this study [28]. Statistically significant difference was defined from the *p* value < 0.05. Statistical analyses were performed using the SPSS program (SPSS version 15, SPSS Inc., Chicago, IL).

## CONCLUSIONS

In summary, ACE and AGT gene polymorphisms directly may have no significant influence on the development of DR in Chinese T1DM patients. However, interaction of hypertension with the RAS polymorphisms might have a role in the vascular complication.
